# *FDXR* is a biomarker of radiation exposure in vivo

**DOI:** 10.1038/s41598-017-19043-w

**Published:** 2018-01-12

**Authors:** Gráinne O’Brien, Lourdes Cruz-Garcia, Matthäus Majewski, Jakub Grepl, Michael Abend, Matthias Port, Aleš Tichý, Igor Sirak, Andrea Malkova, Ellen Donovan, Lone Gothard, Sue Boyle, Navita Somaiah, Elizabeth Ainsbury, Lucyna Ponge, Krzysztof Slosarek, Leszek Miszczyk, Piotr Widlak, Edward Green, Neel Patel, Mahesh Kudari, Fergus Gleeson, Volodymyr Vinnikov, Viktor Starenkiy, Sergii Artiukh, Leonid Vasyliev, Azfar Zaman, Christophe Badie

**Affiliations:** 1 Centre for Radiation, Chemical and Environmental Hazards, Public Health England, Oxfordshire, United Kingdom; 2 Bundeswehr Institute of Radiobiology, Munich, Germany; 3 Department of Radiobiology, Faculty of Military Health Sciences in Hradec Králové, University of Defence in Brno, Hradec Králové, Czech Republic; 4 Biomedical Research Centre, Hradec Králové University Hospital, Hradec Králové, Czech Republic; 5 Department of Oncology & Radiotherapy and 4th Department of Internal Medicine - Hematology, University Hospital, Hradec Králové, Czech Republic; 6 Department of Hygiene and Preventive Medicine, Faculty of Medicine in Hradec Králové, Charles University, Hradec Králové, Czech Republic; 7 Centre for Vision, Speech and Signal Processing, University of Surrey, Guildford, GU2 7TE United Kingdom; 8 Institute of Cancer Research/Royal Marsden NHS Foundation Trust, Downs Road, Sutton, SM2 5PT United Kingdom; 9 Maria Sklodowska-Curie Institute – Oncology Center, Gliwice Branch, Gliwice, Poland; 10 Department of Radiology, Churchill Hospital, Oxford, United Kingdom; 11 Grigoriev Institute for Medical Radiology, National Academy of Medical Science, Kharkiv, Ukraine; 12 Department of Cardiology, Freeman Hospital and Institute of Cellular Medicine, Newcastle University, Newcastle-upon-Tyne, Newcastle, United Kingdom

## Abstract

Previous investigations in gene expression changes in blood after radiation exposure have highlighted its potential to provide biomarkers of exposure. Here, FDXR transcriptional changes in blood were investigated in humans undergoing a range of external radiation exposure procedures covering several orders of magnitude (cardiac fluoroscopy, diagnostic computed tomography (CT)) and treatments (total body and local radiotherapy). Moreover, a method was developed to assess the dose to the blood using physical exposure parameters. FDXR expression was significantly up-regulated 24 hr after radiotherapy in most patients and continuously during the fractionated treatment. Significance was reached even after diagnostic CT 2 hours post-exposure. We further showed that no significant differences in expression were found between *ex vivo* and *in vivo* samples from the same patients. Moreover, potential confounding factors such as gender, infection status and anti-oxidants only affect moderately FDXR transcription. Finally, we provided a first *in vivo* dose-response showing dose-dependency even for very low doses or partial body exposure showing good correlation between physically and biologically assessed doses. In conclusion, we report the remarkable responsiveness of FDXR to ionising radiation at the transcriptional level which, when measured in the right time window, provides accurate *in vivo* dose estimates.

## Introduction

In the last decade or so, there has been a large international research effort to develop new biomarkers of radiation exposure allowing rapid and high-throughput dose estimation. Sixteen years ago, Amundson *et al*.^1^ suggested that relative levels of gene expression in peripheral blood cells may provide an estimation of environmental radiation exposures. Since then, the discovery and assessment of new genes for which transcriptional expression can be modulated by DNA damage in general and indeed ionizing radiation has increased dramatically.

Large scale studies were performed utilising microarrays and quantitative polymerase chain reaction (QPCR) to scan the genome for radiation responsive genes over a range of doses and time-points^1–5^. A panel of suitable genes which are responsive in human blood was established in *ex vivo* irradiated human peripheral blood mononuclear cells (PBMCs)^6–8^ [5–7] as well as whole blood^9,10^ [8, 9], *in vivo* irradiated patient samples^11,12^ and in internally irradiated patients treated with therapeutic radionuclides^13^ with *FDXR* (Ferredoxin Reductase) emerging as one of the most accurate genes for providing dose estimates due to its dose-dependent transcriptional up-regulation^9,14–17^. A post-radiation variation in the expression of *FDXR* was, to our knowledge, for the first time reported by Jen *et al*.^18^. The FDXR flavoprotein transfers electrons from NADPH to mitochondrial cytochrome P450 enzymes^19^. It is regulated by p53 and it has recently been shown that FDXR and p53 are mutually regulated by a FDXR-p53 loop via iron homeostasis. Mechanistically, the literature is relatively scarce on FDXR but very recently, it was shown to regulate several components of the iron pathway one of which, IRP2, negatively regulates p53 expression^20^. Unsurprisingly, FDXR is also involved in ROS (Reactive Oxygen Species) associated apoptosis^21,22^. *FDXR* has been reported as the only gene differentially expressed following exposure of cells to 6 out of 7 anti-cancer drugs treatment tested^23^ and a transcriptional up-regulation of *FDXR* can be considered as a universal response to DNA damage.

After exposure to ionising irradiation, *FDXR* produces one of the highest levels of fold changes in the blood^9^. This high level of expression, combined with a relatively low level of endogenous expression and variability among individuals, allows for easy discrimination of high doses from low doses^9^. This fact, along with the linear increases in expression observed at low and high doses made it an attractive gene for assessing exposure in blood. Several *ex vivo* studies have validated *FDXR* as a sensitive and reliable gene for assessing radiation dose in three large scale studies involving 9 laboratories from multiple countries^14,16,17^. Due to the scarcity of *in vivo* irradiated human blood samples, *ex vivo* irradiated samples have previously been used to construct a calibration curve to investigate in the vivo transcriptional response to radiation. In a NATO (North Atlantic Treaty Organization), led exercise, a blood sample from one individual was irradiated *ex vivo* and sent to 7 different laboratories for analysis with *FDXR* being favored as the gene of choice by 4 of the laboratories^16^. Gene expression dose estimates that were produced without protocol standardisation, although not as accurate as the gold standard dicentric assay, showed that gene expression is a potentially useful technique for triage due to the speed and throughput of the assay^23^. In 2015, a RENEB (Realising the European Network of Biodosimetry) interlaboratory comparison exercise was performed, this time using *ex vivo* irradiated blood from 12 donors in total and for the first time, also *in vivo* irradiated blood from 4 prostate cancer patients before and after radiotherapy. Once again *FDXR* was seen to be the best gene for dose estimation for *ex vivo* irradiated blood and, it was confirmed for the first time that it could distinguish blood samples from patients prior to radiation exposure from post-irradiated patient blood samples although irradiation was limited to a localised area^17^ and that the dose to the blood was estimated to be extremely low compared to doses tested *ex vivo* so far at the exception of Manning *et al*. 2013^9^.

Other factors which can potentially affect its expression such as age, gender, smoking, environmental factors or infection have yet to be studied for the gene *FDXR*; these potential confounding factors may change endogenous transcriptional level. Lipopolysaccharides (*LPS*) are commonly used to mimic a bacterial infection and these molecules elicit immune responses; therefore the effect of LPS as a stress factor has been investigated as a confounding factor in DNA damage response genes^24^. LPS exposure was seen to up-regulate *CDKN1A*, *BBC3* and *FDXR* gene expression even without radiation exposure and in their specific conditions, a fold of change of 1.5 was found compared to the control for *FDXR*, and the increase in expression was the lowest observed amongst the three genes. This requires further investigation in case of complex (e.g. inflammation stress), multiple exposure scenarios.

Previous work has investigated gene expression in *in vivo* irradiated cancer patients^2,17,25^ and medical workers^26^, however the majority of experiments involve *ex vivo* irradiated samples. The few studies which have involved *in vivo* irradiated patients have investigated the response using a panel of genes. Several papers have emphasized the need to develop a signature of radiation exposure based on the modification of a group of genes^9,27^, but the recent data obtained in blood exposed *ex vivo* and *in vivo* in samples from prostate cancer patients treated by radiotherapy suggested that *FDXR* was to date the best ‘stand-alone’ biomarker of ionising radiation exposure and suitable for biological dosimetry in human blood^17^. In this study, using blood samples collected from humans exposed *in vivo* to a very large range of doses covering several orders of magnitude, we provide for the first time, physical and biological dose estimates using *FDXR* gene expression. Here we confirm the validity of the data obtained mostly *ex vivo* so far by comparing blood samples from the same patient irradiated *ex vivo* and *in vivo*, and demonstrate the remarkable sensitivity of *FDXR* in whole blood exposed *in vivo*, validating it as a biomarker of radiation exposure.

## Results

### FDXR Expression

**Figure 1:**
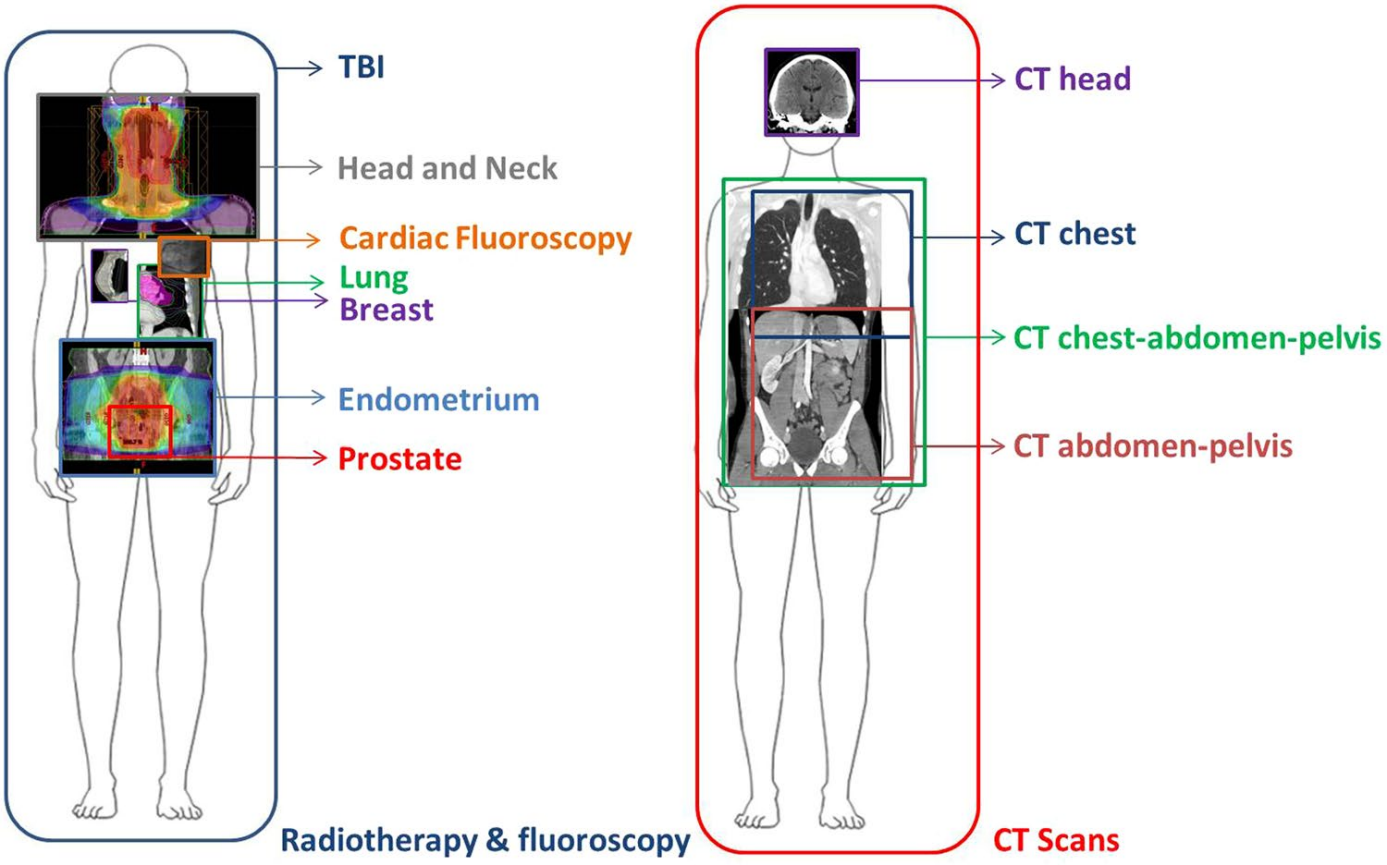
Graphic representation of exposure areas of each patient sub-group; Total body irradiation (TBI), head and neck cancer, cardiac fluoroscopy, lung cancer, breast cancer, endometrial cancer and prostate cancer (left) and computed tomography (CT) sub-groups of head chest, chest-abdomen-pelvis and abdomen-pelvis (right). Human body outlines were obtained from wikimedia. Human body outline image by Priyanka250696 (Own work) [CC BY-SA 4.0 (https://creativecommons.org/licenses/by-sa/4.0)], via Wikimedia Commons.

**Figure 2:**
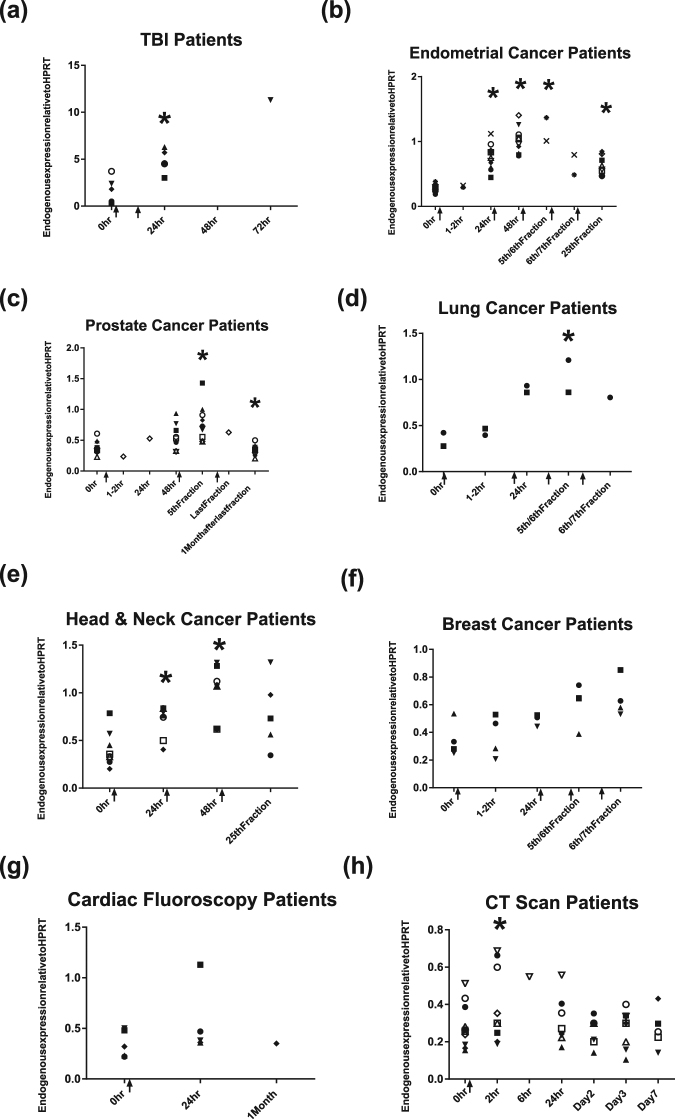
Transcriptional FDXR gene expression changes in peripheral blood samples from (**a**) TBI patients (6 patients), (**b**) endometrial cancer radiotherapy patients (12 patients), (**c**) prostate cancer radiotherapy patients (10 patients), (**d**) lung cancer radiotherapy patients (2 patients), (**e**) head and neck cancer radiotherapy patients (8 patients), (**f**) breast cancer radiotherapy patients (4 patients), (**g**) patients undergoing cardiac fluoroscopy (5 patients) and (**h**) patients undergoing diagnostic CT (10 patients), before radiation exposure and at different times post-exposure with each symbol representing one patient. Expression is relative to *HPRT* gene. Statistical analyses were performed in log transformed data. Significant differences (Paired-T-test, p ≤ 0.05) with the control were indicated with an asterisk (*). Arrows indicate time of irradiation.

A graphic representation of exposure areas of each patient sub-group is presented in Fig. 1; the dose delivered per fraction and the physical estimated dose to the blood are shown in Table 1. *FDXR* expression in patient samples exposed to either a high total body irradiation (TBI) or a range of low doses was assessed. In general, for radiotherapy patients, up-regulation after the first fraction could be detected in most cases and this up-regulation was maintained during the course of radiotherapy, lasting weeks for some patients (Fig. 2, Supplementary Table S1). A dose effect was clear with patients receiving the highest doses to the blood (TBI), showing the highest expression of *FDXR* and conversely, the lowest expression was seen in blood from patients who received the lowest dose to the blood.

**Table 1:** Patient number with associated symbol, gender, age, physical dose fractions, calculated estimated dose to the blood, total number of fraction and overall radiotherapy treatment time for TBI, endometrial cancer, head and neck cancer, breast cancer, prostate cancer, lung cancer patients, diagnostic computed tomography (CT) and cardiac fluoroscopy patients.

Category	Patient Number & Symbol	Gender	Age	Fraction Dose	FDXR 24 hr Fold Change	Estimated dose to the blood per fraction (mGy)	Total No. of Fractions	Overall Radiotherapy Treatment Time
**TBI Patients**	1 ●	Female	23	1.575 Gy	8.5	3.2 Gy	2	8 hr
	2 ■	Male	39	1.5 Gy	11.7	3 Gy	2	8 hr
	3 ▲	Female	48	2 Gy	11.4	4 Gy	2	8 hr
	4 ▼	Male	27	2 Gy	4.7	12 Gy	6	56 hr
	5 ♦	Male	28	2 Gy	3.2	4 Gy	2	8 hr
	6 ◯	Male	31	2 Gy	1.2	4 Gy	2	8 hr
**Endometrial Cancer Patients**	1 ●	Female	75	1.8 Gy	3	170	25	5 Weeks
	2 ■	Female	64	1.8 Gy	2	210	25	5 Weeks
	3 ▲	Female	79	1.8 Gy	1.8	190	25	5 Weeks
	4 ▼	Female	65	1.8 Gy	3	200	25	5 Weeks
	5 ♦	Female	71	1.8 Gy	2.4	190	25	5 Weeks
	6 ◯	Female	57	1.8 Gy	3.9	160	25	5 Weeks
	7 □	Female	69	1.8 Gy	2.8	220	25	5 Weeks
	8 △	Female	78	1.8 Gy	2.2	160	25	5 Weeks
	9 ▽	Female	74	1.8 Gy	2.9	170	25	5 Weeks
	10 ◇	Female	74	1.8 Gy	2.8	190	25	5 Weeks
	11 ✴	Female	71	1.8 Gy	2.2	190	25	5 Weeks
	12 X	Female	69	1.8 Gy	3.7	140	25	5 Weeks
**Prostate Cancer Patients**	1 ●	Male	65	7.25 Gy	1.5	100	5	1.5 Week
	2 ■	Male	69	7.25 Gy	1.4	140	5	1.5 Week
	3 ▲	Male	67	7.25 Gy	1.9	120	5	1.5 Week
	4 ▼	Male	68	7.25 Gy	2.2	130	5	1.5 Week
	5 ♦	Male	68	7.25 Gy	1.6	130	5	1.5 Week
	6 ◯	Male	64	7.25 Gy	1.2	180	5	1.5 Week
	7 □	Male	74	7.25 Gy	1.3	90	5	1.5 Week
	8 △	Male	67	7.25 Gy	1.7	210	5	1.5 Week
	9 ▽	Male	52	7.25 Gy	1.2	150	5	1.5 Week
	10 ◇	Male	65	3 Gy	1.5	50	20	4 Weeks
**Lung Cancer Patients**	1 ●	Male	86	2.75 Gy	2.2	110	20	4 Weeks
	2 ■	Male	65	2.75 Gy	3.1	80	20	4 Weeks
**Head and Neck Cancer Patients**	1 ●	Female	52	2 Gy	2.8	80	25	5 Weeks
	2 ■	Male	57	2 Gy	1.3	110	25	5 Weeks
	3 ▲	Male	81	2 Gy	1.4	90	25	5 Weeks
	4 ▼	Male	79	2.1 Gy	1.7	70	25	5 Weeks
	5 ♦	Male	55	2.1 Gy	1.6	110	25	5 Weeks
	6 ◯	Male	51	2.1 Gy	2	90	25	5 Weeks
	7 □	Male	52	2 Gy	1.4	80	25	5 Weeks
	8 △	Male	66	2 Gy	2.3	90	25	5 Weeks
**Breast Cancer Patients**	1 ●	Female	66	2.67 Gy	1.5	60	15	3 Weeks
	2 ■	Female	62	2.67 Gy	1.9	70	15	3 Weeks
	3 ▲	Female	69	2.67 Gy	1	70	15	3 Weeks
	4 ▼	Female	73	2.67 Gy	1.8	70	15	3 Weeks
**Cardiac Fluoroscopy Patients**	1 ●	Male	63	28 mSv	2.1	—	1	N/A
	2 ■	Male	78	76 mSv	2.4	—	1	N/A
	3 ▲	Male	52	17 mSv	1.6	—	1	N/A
	4 ▼	Female	78	9 mSv	0.8	—	1	N/A
	5 ♦	Male	80	19 mSv	1.1	—	1	N/A
**CT Patients**	1 ●	Female	47	13 mGy	N/A	3.9	1	N/A
	2 ■	Female	59	13 mGy	1	3.7	1	N/A
	3 ▲	Female	68	11 mGy	1	6.6	1	N/A
	4 ▼	Female	67	13 mGy	1.3	3.8	1	N/A
	5 ♦	Male	54	8.5 mGy	0.7	2.9	1	N/A
	6 ◯	Female	74	7.9 mGy	0.6	5.2	1	N/A
	7 □	Female	69	16 mGy	1.1	11.3	1	N/A
	8 △	Male	75	26 mGy	N/A	12.2	1	N/A
	9 ▽	Female	37	36 mGy	1.1	11.9	1	N/A
	10 ◇	Male	78	30 mGy	1.5	20.9	1	N/A

In detail, *FDXR* expression showed an increase in expression in TBI patients 24 hr after a cumulative dose of 3 or 4 Gy with some variability evident among the TBI patients and was still detectable at 72 hours in the only patient from whom a blood sample was taken (Fig. 2a). The influence of tumour presence on FDXR expression in blood white blood cells has also been investigated. FDXR showed similar levels of expression in pre-exposure cancer samples as in normal donor samples (data not shown). To be noted, three TBI patients showed an increased level of FDXR expression in pre-exposure samples, possibly due to the very ill state of the patients who require TBI (data not shown). *FDXR* expression in endometrial cancer patients showed a significant increase in expression in all 10 patient samples evident from 24 hr after the first fraction (Fig. 2b), detectable at later time points during the course of treatment and until its end. At the earliest time point (1–2 hr) *FDXR* expression levels did not differ to pre-exposure samples, however, data was only available for two patients at this time-point and there is uncertainty (between 1 and 2 hour post exposure). 24 hr after the second fraction, at 48 hr, a further increase in *FDXR* expression was again evident. At the end of radiotherapy treatment at 5 weeks, *FDXR* expression decreased from the expression at 48 hr but was still up–regulated in comparison to controls. Prostate cancer patients receiving radiotherapy showed an increase in *FDXR* expression during the course of radiotherapy, becoming significant after the 5th fraction (Fig. 2c). The first time-point to show an increase in expression was at 48 hr, but only one patient sample was available at 1–2 hr and 24 hr, thus not allowing us to reach any conclusion. Samples were also taken 1 month after the last fraction and interestingly, *FDXR* levels significantly dropped since returning to pre-exposure levels, although changes are small. *FDXR* expression in lung cancer patients during radiotherapy showed an increase in *FDXR* expression 24 hr after the first fraction and remained significantly up-regulated for the duration of radiotherapy, 24 hr before the 5th or 6th fraction (Fig. 2d). Head and neck cancer patients received on average a physically calculated blood dose of 92 mGy, which was similar to the lung cancer patients (Fig. 2e). 24 hr after the first fraction, a significant increase in *FDXR* expression was evident in all patients, which was sustained at 48 hr in all but one patient. By the end of the treatment, *FDXR* was still up-regulated in 4 out of the 5 patient samples at this time-point. The expression of another gene expressed after irradiation, CDKN1A, was also investigated, however, its expression at 24 hr after ionising radiation was comparable to control samples (data not shown) and so FDXR was the only gene of interest in this study. *FDXR* expression in breast cancer patients, who received a lower mean calculated dose to the blood of 67 mGy per fraction, was clearly increased 24 hr after the first fraction in 3 out of 4 patients and this increase continued for the rest of the radiotherapy although significance couldn’t be reached (p = 0.057) probably due to the limited number of patients in this group (Fig. 2f).

Patient samples that received the lowest doses of radiation, were cardiac fluoroscopy patients (Fig. 2e) and patients undergoing diagnostic computed tomography (CT) (Fig. 2f). There was a slight up-regulation of *FDXR* at 24 hr for cardiac fluoroscopy patients, with one patient showing a 2 fold increase in expression, and at 2 hr and 24 hr for diagnostic CT patients. It is worthwhile stating that 4 out of the 5 fluoroscopy patient samples at 24 hr were up-regulated in comparison to their pre-exposure controls. For diagnostic CT patients, and amazingly, considering the physically calculated dose to the blood, 6 out of 8 samples were up-regulated and this small increase in the transcriptional expression was significant at 2 hr but was no longer significant at 24 hr and returned to pre-exposure levels by day 2.

### *Ex Vivo* Dose Estimation Curve

**Figure 3:**
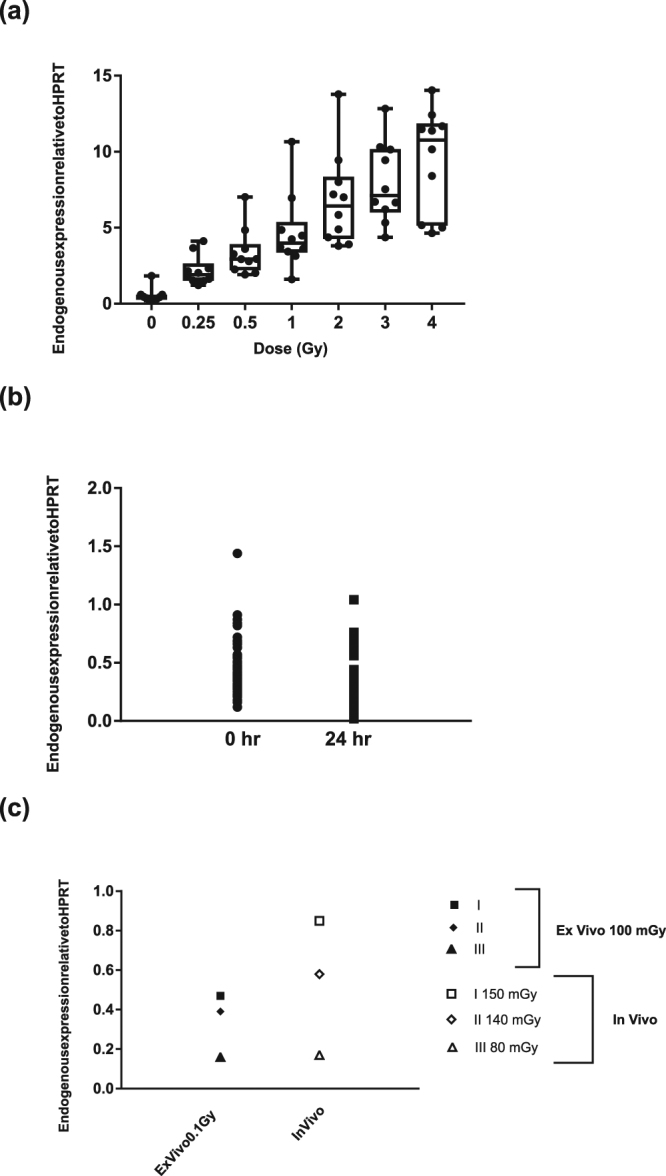
Comparison of *FDXR* expression in blood in *in vivo* and *ex vivo* irradiated samples. (**a**) The box plot shows MQRT-PCR gene expression changes in *FDXR* expression in *ex vivo* irradiated blood from 10 normal healthy human blood donors 24 hr after irradiation with 0, 0.25, 0.5, 1, 2, 3 and 4 Gy x-rays. Expression is relative to *HPRT* gene. (**b**) *FDXR* expression at 0 hr in 82 healthy human donors and in blood kept *ex vivo* at 37 °C for 24 hr in 39 healthy human donors. (**c**) Comparison of *FDXR* expression at 24 hr in blood irradiated *ex vivo* and *in vivo* from three donors. For *ex vivo* samples, FDXR expression is presented 24 hr after *ex vivo* irradiation with 100 mGy in blood samples taken pre-exposure from the same patients. For *in vivo* samples, FDXR expression is presented 24 hr after the first radiotherapy fraction dose where cancer patients were irradiated with blood doses of 150 mGy for patient I, 140 mGy for patient II and 80 mGy for patient III.

An *ex vivo* irradiated calibration curve from 10 healthy human blood donors was obtained to provide biological dose estimates (Fig. 3a, Supplementary Table S2). As the calibration curve was obtained 24 hours post exposure, we compared a possible change in expression in unexposed blood kept *ex vivo* at 37 °C for 24 hr; *FDXR* expression from 82 donors at 0 hr and from 39 donors at 24 hr. Although a slight decrease in expression was evident after 24 hr incubation at 37 °C in comparison to samples at 0 hr (0 hr − 0.41, 24 hr - 0.36), this was not significant (Fig. 3b, Supplementary Table S2). As physiological conditions are fundamentally different *in vivo* and *ex vivo*, we wanted to validate the results found *ex vivo*, and for the first time to our knowledge, blood samples from the same radiotherapy patients were taken before treatment and 24 hr after radiotherapy with a physically calculated blood dose of 150 mGy (patient I), 140 mGy (patient II) and 80 mGy (patient III) (Fig. 3c, Supplementary Table S2). Blood samples from the three patients before the first radiotherapy dose fraction were also irradiated with 100 mGy *ex vivo*. Remarkably, *FDXR* expression in *in vivo* irradiated patient samples with blood doses of 150 mGy and 140 mGy had higher *FDXR* levels, of 1.8 fold and 1.5 fold respectively, in comparison to their 100 mGy *ex vivo* irradiated samples. Patient III who had an *in vivo* irradiated dose of 80 mGy had similar *FDXR* expression at 0.17 to its 100 mGy *ex vivo* irradiated sample at 0.16.

### Inter-laboratory Comparison

**Figure 4:**
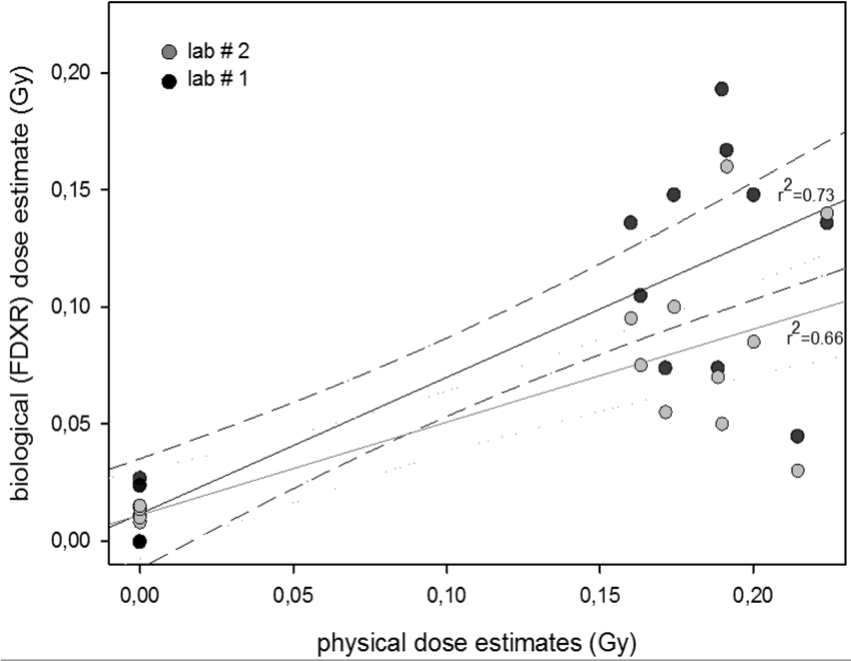
Comparison of dose estimates from 2 independent laboratories of blood samples from endometrial cancer patients before radiotherapy and 24 hr after the first fraction of 1.8 Gy.

In order to be confident in our results and the reliability of the biologically calculated dose estimates using *ex vivo* obtained calibration curves for *FDXR*, dose estimates were provided by two independent laboratories for the endometrial radiotherapy treated patients set at 0 hr and 24 hr as shown in Fig. 4. Both laboratories provided comparable sets of data with no significant differences in dose estimates (p = 0.1859). Lab 1 obtained dose estimates ranging from 50–190 mGy, while lab 2 provided dose estimates ranging from 30–140 mGy.

### Modulation of FDXR response to radiation by LPS and curcumin

**Figure 5:**
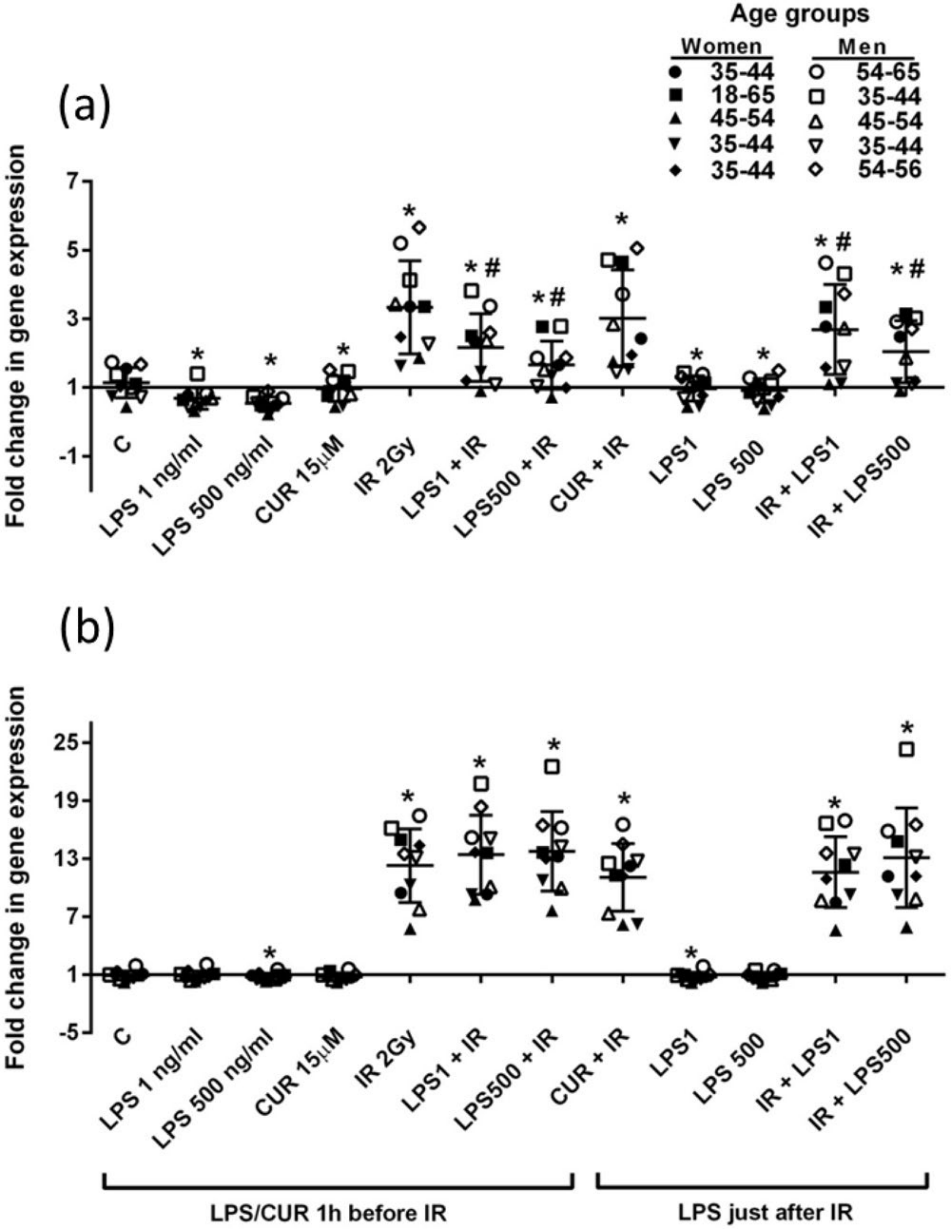
Gene expression of *FDXR* in human blood irradiated and/or stimulated with LPS and curcumin *ex vivo*. Blood from 10 donors was incubated with two different concentration of LPS (1 or 500 ng ml-1) or curcumin (15 µM) 1 hr before irradiation (2 Gy) or just after irradiation (only for LPS). Transcriptional expression of FDXR was analysed at 2 hr (**a**) and 24 hr post-irradiation (**b**). Data are shown as mean ± SD (n = 10, black symbols indicate five women and white symbols five men). Statistical analyses were performed on log transformed data. Significant differences (Paired-T-test, p ≤ 0.05) with the control were indicated with an asterisk (*) and differences with IR with a hash (#) (only for IR groups).

In order to assess the role of three potential confounding factors, the effect of an inflammatory stimulus and an anti-inflammatory agent in the modulation of radiation induced *FDXR* upregulation was tested *ex vivo* in human blood from male and female healthy donors (Fig. 5, Supplementary Table S3). We used lipopolysaccharides (LPS), which are endotoxins, and elicit strong immune responses in animals. LPS modulation of *FDXR* expression was observed alone and combined with exposure to ionizing radiation. The results showed a downregulation of *FDXR* expression at low and high concentrations of LPS (1 and 500ng/ml). When LPS was present before and after IR, LPS counteracts the IR upregulation of *FDXR* but only after a short time post-stimulation (2 hr post-irradiation). However, this down-regulatory effect of LPS on IR-*FDXR* induction is lost after 24 hr post-exposure. LPS alone still exerts a slight decrease on *FDXR* expression at 24 hr.

With regard to the effect of curcumin on *FDXR* gene expression, curcumin slightly downregulated its expression only after a short incubation time (3 hr), but did not modulate *FDXR* induction mediated by IR. At 24 hr, curcumin did not show any regulatory effect on *FDXR* gene expression alone or combine with IR exposure. In terms of gender, there were no significant differences found between men and women for any of the *FDXR* expression responses to LPS, curcumin and IR.

### Biological Dose Estimation

**Figure 6:**
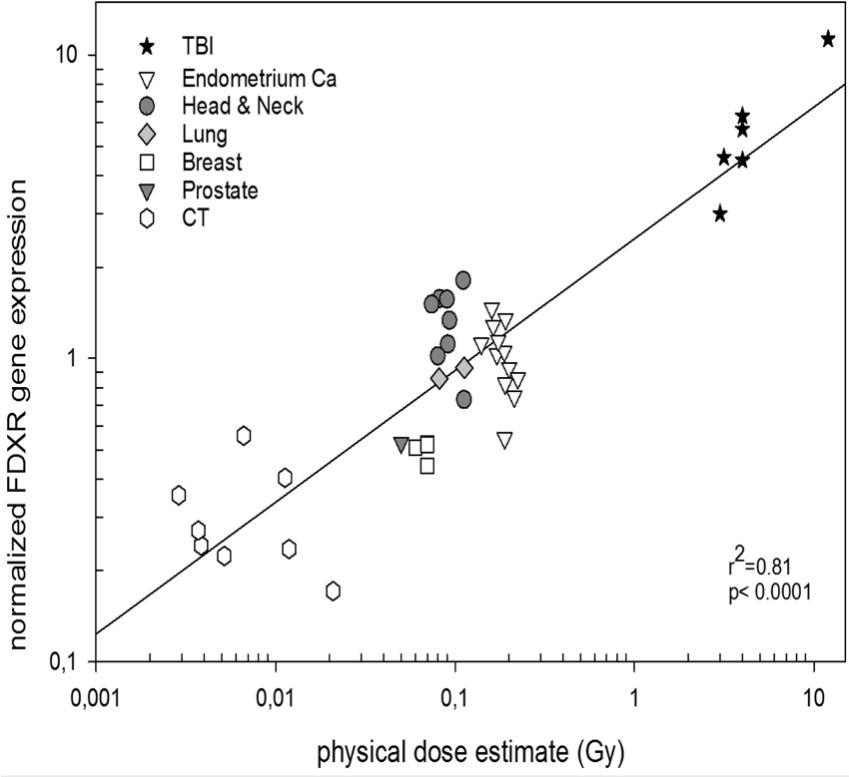
*FDXR* mRNA endogenous expression in blood samples from patients exposed to a variable range of radiation doses. Patient groups include TBI, endometrial cancer, head and neck cancer, breast cancer, prostate cancer, lung cancer and patients undergoing diagnostic CT. All samples were taken at 24 hr after radiation exposure. The data are represented on a log transformed scale with a linear fit and R^2^ and p values plotted.

In order to obtain *in vivo* dose-response in humans, the endogenous expression of *FDXR* at 24 hr in all patient groups was plotted with the corresponding physically estimated dose. There was a significant linear relationship between normalized *FDXR* CT-values and the physical dose estimate (Fig. 6). The lowest level of expression was found in the diagnostic CT patients’ group which received the lowest doses while the highest level of expression was in the TBI patients who received the highest dose. In the low dose range (between 2.9 and 220 mGy) an increasing expression of *FDXR* was evident from the diagnostic CT patients, increasing further for the breast, prostate, lung, endometrial and head and neck cancer patients with increasing dose.

**Figure 7:**
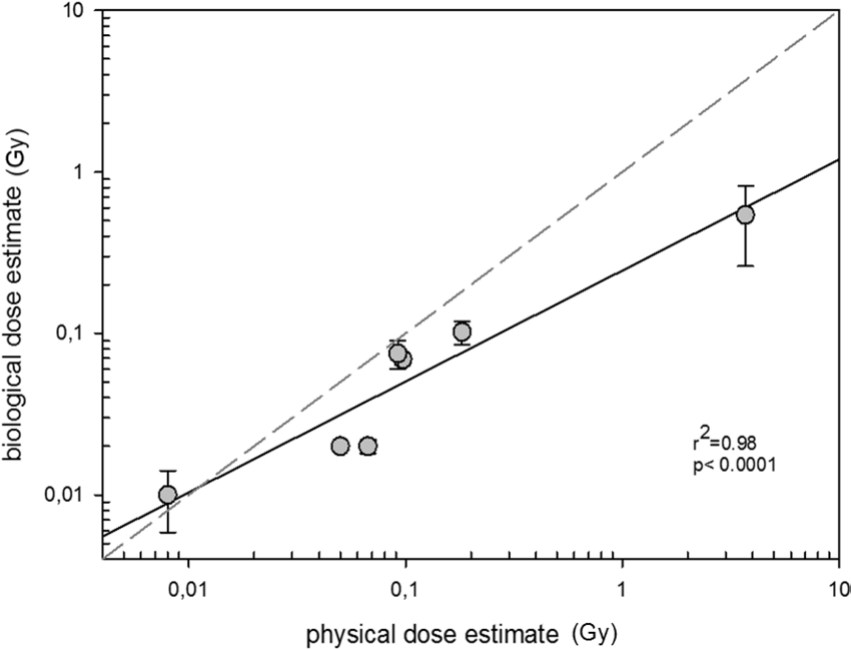
*FDXR* calculated dose estimates in blood samples from cancer radiotherapy patients and diagnostic CT procedures exposed to a variable range of radiation doses. Patient groups include TBI, endometrial cancer, head and neck cancer, breast cancer, prostate cancer, lung cancer and diagnostic CT patients. All samples were taken at 24 hr after radiation exposure. The data represent the standard error of mean (SEM) of the biological dose estimates (y axis) and of the physical dose estimates (x axis) on a log transformed scale with a linear fit and R^2^ and p values plotted.

In order to compare both biological and physical dose estimates, calculated respectively using *FDXR* gene expression in cancer radiotherapy patients at 24 hr and the *ex vivo* calibration curve and calculated by use of algorithms using specific parameters, blood volume, weight and radiotherapy details, a regression analysis was performed (Fig. 7). A calibration curve generated on *ex vivo in vitro* irradiated peripheral blood was employed for biological dosimetry purposes which lead to a significant linear dose relationship of the biological dose estimate (based on *FDXR* gene expression) and the corresponding physical dose estimate over a dose range of 10–3500 mGy or 3.5 log-scales (Fig. 7). In particular the fractionated high total body exposures were converted into an about 6-fold lower dose estimate based on biological dosimetry, while lower and single exposures to the patient converted into biological dose estimates comparable to the physical dose estimates. This leads to a deviation of the linear regression from an ideal association (Fig. 7, dashed line) of biological dosimetry with physical dosimetry at high doses.

Overall, the results show that both independent dose estimates are in good agreement, as there is a linear increase in biological dose estimate with increasing physical dose estimate for all patients (R^2^ = 0.98 and p < 0.0001) and in the low dose range (R^2^ = 0.85 and p = 0.0079).

## Discussion

*FDXR* is a gene for which the function was relatively unclear until a recent publication in July 2017 by Zhang *et al*.^20^ showing that *FDXR* is an essential gene, critical for p53-dependent tumour suppression via iron regulatory protein 2. In the past few years, *FDXR* transcriptional gene expression has been reported by us and others in *ex vivo* studies as a biomarker of radiation exposure in blood leukocytes. This was promising as gene expression monitoring in blood samples is minimally invasive, involves a very quick processing time, is high throughput and requires minimal expertise. Nevertheless, studies reported so far have been carried out in blood samples irradiated *ex vivo*, only mentioned *in vivo* in one recent publication^17^. Here, in order to validate *FDXR* as a biomarker of radiation exposure *in vivo*, we carried out an extensive study using a panel of blood samples collected across several hospitals in Europe from patients undergoing either total body exposure, radiotherapy for a range of malignancies or other procedures involving radiation exposure at much lower doses such as CT scans and fluoroscopy.

Expression of *FDXR* was seen to be up-regulated after the first fraction of radiotherapy and remained up-regulated with multiple radiation exposures until the last fraction of radiotherapy at varying doses (Fig. 2). Moreover, after a cumulative dose of either 3 Gy or 4 Gy for TBI patients, a strong up-regulation in *FDXR* expression was seen in all patients reaching an 11 fold up-regulation for patient 4 who received a cumulative dose of 12 Gy, although variation in expression among the TBI patients was evident. The fact that TBI patients have extremely severe underlying illnesses, leukaemia in these cases, may be a factor in this variation as gene expression was monitored in blood of patients who have often a very low blood count and which may contain leukaemic cells. Compared to TBI patients, endometrial cancer patients received a significantly lower blood dose fraction of about 190 mGy but still exhibited a strong *FDXR* up-regulation at all time-points. For the remaining cancer patient groups (prostate, lung, head and neck, breast), fractionated radiation exposure resulted in consistent *FDXR* up-regulation in all groups.

Following other medical procedures, cardiac fluoroscopy and diagnostic CT patients, blood samples were also collected to cover exposure to very low doses of radiation with calculated blood doses as low as 20 mGy and below for diagnostic CT patients. Although the calculated effective dose for cardiac fluoroscopy patients is subject to significant uncertainty, the patient who received the highest effective dose of 76 mSv (patient 2), also produced a 2 fold increase in *FDXR* expression at 24 hr while remaining patients who received doses below 28 mSv showed only slight up regulation. *FDXR* expression appeared generally unchanged for cardiac fluoroscopy and diagnostic CT patients, however, a 1.3 to 1.7 fold up-regulation was still evident in some patients after exposure with a significant up-regulation at 2 hr in diagnostic CT patients. This was surprising as we previously showed that the level of transcriptional up-regulation is higher at 24 hours post exposure compared to 2 hours in *ex vivo* exposed blood^9^. This is possibly due to the very low dose used whose effect is more pronounced and peaked earlier (at 2 hr_ and does not last for 24 hr. It is difficult to conclude as for higher doses, only 2 patient samples were obtained 2 hr after the first radiotherapy fraction for endometrial cancer with one sample showing an up-regulation of *FDXR* and one patient showing a down-regulation. More patient samples exposed to higher and lower doses at 2 hr would be required to confirm these data. Interestingly, gamma H2AX foci blood samples taken 5 minutes after chest-abdominal-pelvic CT were significantly higher (corresponded to a mean radiation dose of 16.4 mGy) compared to background levels before CT taken^28^. This significance was lost at later time points. It is possible that the up-regulation triggered via the DNA damage response (ATM/CHEK2/p53) vanished if the very low numbers of DNA double-strand breaks are rapidly repaired^29^.

Further investigation into the effect of high and low doses on *FDXR* expression over time *in vivo* would be interesting although practicality, in obtaining regular blood samples from radiotherapy patients for a detailed time-course of *FDXR* would be difficult.

We had previously studied *FDXR* expression in blood *ex vivo* but did not compare the expression with *in vivo* samples. Assessment of the *ex vivo* storage conditions at 37 °C showed a decreased expression of *FDXR*, which was however not significant, while comparison of *FDXR* expression in *ex vivo* and *in vivo* irradiated samples from the same patients showed similar levels of *FDXR* expression over the period of time where it was followed. Overall these results show how representative the *ex vivo* irradiation is of the *in vivo* response and validates previous experiments using *ex vivo* irradiated blood. Importantly, blood exposed *ex vivo* and *in vivo* to similar doses lead to comparable levels of *FDXR* up-regulation.

In order to further validate our findings, samples were sent to a collaborative laboratory in Germany (Bundeswehr Institute of Radiobiology, Munich*)*. Both labs provided dose estimates for endometrial samples 0 hr and 24 hr after the first mean fraction dose to the blood of 190 mGy. As evident in Fig. 4, the inter-lab comparison generally showed that the estimates were very similar although Lab 1 estimates were often slightly higher than those of Lab 2. This could be due to the protocols which are specific to each laboratory. Nevertheless, dose estimates were overall similar for both labs illustrating how robust the transcriptional up-regulation of *FDXR* is to provide similar dose estimates.

An inflammatory stimulus (LPS-induced) showed a downregulation of *FDXR* expression at 2 hr and also a reduction of its upregulation mediated by IR. This regulatory action of LPS is practically lost at 24 hr, probably due to neutralization of LPS inflammatory activity by plasma components^30,31^. A previous study showed a down regulatory effect of LPS in *FDXR* at 24 hr in human blood exposed to 2 Gy X-rays^24^. These differences could be attributed to different experimental setups after irradiating the blood, such as blood dilution with media during the incubation periods post-exposure for example. The present results indicate that LPS, in the range of concentration used here, can modulate and counteract IR action on *FDXR* transcriptional activation shortly after exposure- but to a lower extend at longer time points post-exposure.

Curcumin has been described to have anti-inflammatory, antioxidant and radioprotective properties^32,33^. In the present study curcumin slightly downregulated *FDXR* expression after 2 hr but did not affect the IR induction of *FDXR*. Curcumin is known to induce p53 expression^34^, the pathway whereby IR regulates *FDXR* expression. However, a higher upregulation of *FDXR* by the combination of IR and curcumin was not observed. This lack of a synergistic effect between IR and curcumin was previously shown in leukocytes exposed to IR and treated with curcumin in genes involved in radiation response^34^. Overall, the role of potential confounding factors affecting *FDXR* transcription such as gender, lipopolysaccharides (LPS) and curcumin only had, albeit sometimes significant, limited interfering effects.

Taken together, the *FDXR* expression 24 hr after radiation exposure in all patient samples can provide biologically estimated doses to the blood. The response was linear with a high R^2^ value of 0.98 for all samples. Importantly however, one patient group, TBI patients, provided biological dose estimates which were much lower than their mean physically estimated blood doses. This discrepancy might reflect the impaired health status and/or the fractionated dose. It also might be interpreted as an indication that biological dosimetry might better integrate individual characteristics (e.g. the individual health status) than employing physical dosimetry. As a whole, the calculated dose estimates for all categories of radiotherapy cancer patients are slightly underestimated. However, it is worth remembering that the radiotherapy calculated dose to the blood is an estimate and must be interpreted with caution. It is very difficult to provide accurate estimates to the blood, with no current standard method for calculation, so the dose estimates could in fact be closer to the true dose.

Albeit the dose to the patient is carefully controlled during the radiotherapy treatment, dose estimation using *FDXR* expression could provide a rapid confirmation of the dose received by patients; this could be particularly relevant during fluoroscopy procedures which last longer and require more fluoroscopy, thus creating greater potential radiation exposure to patients and clinicians^35–37^.

Hence the monitoring of the transcriptional expression of the gene *FDXR* in human blood in a specific window of time allows assessment of *in vivo* radiation exposure with good accuracy. This is remarkable especially in the case of partial body exposures and low dose exposure, hence providing a diagnostic marker allowing rapid dose estimates and capable of triage of potentially radiation exposed individuals in case of a nuclear incident, an accidental irradiation or a potential terrorist attack involving a so called dirty bomb that could cause acute and chronic radiation toxicity^12,38^. Moreover, we compared physical and biological dose estimation to the blood with *FDXR* data and found a good correlation.

In conclusion, we report that *FDXR* shows a remarkable sensitivity to ionising radiation exposure over a large range of doses covering several log in human leukocytes *in vivo*, thus demonstrating that *FDXR* is a stand-alone, possibly unique (as it stands) biomarker of radiation exposure in humans. *FDXR* provides accurate dose estimations even after low doses of radiation and could be useful, not only for biodosimetry purposes, but also, alone or in combination with other genes, to allow an assessment of the likely individual radiation responses in terms of early and late normal tissue reactions to the radiation treatment hence participating to the development of personalized medicine.

## Materials and Methods

### Patient Blood Collection

The collection of blood samples from healthy blood donors was carried out with informed consent in accordance with the ethical approval of the West Midlands - Solihull Research Ethics Committee (REC 14/WM/1182) at CRCE, Oxfordshire. The collection of blood samples from two TBI (Fractionated, high-dose total body irradiation (TBI) used to myeloablate and immune suppress patients undergoing hematopoietic stem cell transplantation., endometrial and head and neck cancer patients was performed at the University Hospital in Hradec Kralove (Czech Republic). This study was carried out in accordance with the recommendations of The Code of Ethics of the World Medical Association - Declaration of Helsinki (approval no: 201401-S15P) with written informed consent from all subjects. All subjects gave written informed consent in accordance with the Declaration of Helsinki. The protocol was approved by the Ethical Committee of University Hospital in Hradec Kralove (Czech Republic). Blood samples from three TBI patients were also obtained from Hospital Na Bulovce, Prague, Czech Republic. The local “Ethics Comitte on Trial on Human Medicine Products” approved this study under the code: 10/02/2017. One TBI patient sample was collected from the Department of Radiation Oncology, Hannover Medical School, Hannover, Germany in collaboration with Department of Hematology, Hemostasis, Oncology and Stem cell Transplantation, Hannover Medical School, Hannover, Germany with ethical approval given by the ethics committee of Hannover Medical School (No. 7272). The collection of blood samples from two endometrial patients, breast cancer patients, one prostate cancer patient and two lung cancer patients was performed at The Royal Marsden and Institute of Cancer Research, Surrey where blood was taken with written informed consent from all subjects and the ethical approval by the Health Research Authority (REC 16/SC/0307). The collection of blood samples from prostate cancer patients was carried out in accordance with the Bioethical Committee in Maria Sklodowska-Curie Institute, Warszaw, approval number 27/2015 from 18/08/2015. The collection of blood samples from cardiac fluoroscopy patients was performed at Freeman Hospital, Newcastle with approval by the NRES Committee North East – York (REC 13/NE/0214). The collection of blood samples from diagnostic CT patients was performed at Churchill Hospital with approval from Berkshire B ethics committee (13/SC/0130). The collection of blood samples from cancer patients for *ex vivo* irradiation comparison was performed at the Grigoriev Institute for Medical Radiology of the National Academy of Medical Science of Ukraine. Each patient gave written informed consent to participate in this study. Patient recruitment, including the volume of blood to be taken from a patient and a sampling scheme, was approved by GIMR Committee of Bioethics and Deontology.

### Patient Radiotherapy Fractions

Blood samples from TBI patients, endometrial cancer patients, prostate cancer patients, lung cancer patients, head and neck cancer patients, breast cancer patients, patients who received cardiac fluoroscopy and patients who received a diagnostic CT were collected into PAXGene tubes before radiotherapy treatment and at different times post-exposure. Blood from TBI patients was taken pre-exposure and 24 hr after the first fraction, except for patient 4, who was sampled after 72 hr. TBI patients received two-consecutive fractions, 1^st^ in the morning and 2^nd^ in the afternoon with an 8 hr delay. Blood from endometrial cancer patients was taken pre-exposure, 24 hr after the first fraction, 24 hr after the second fraction and 24 hr after the 25^th^ fraction. For 2 endometrial cancer patients blood was taken pre-exposure, ½ hr after the first fraction, 24 hr after first fraction, 24 hr before the 5^th^/6^th^ fraction and 24 hr before the last fraction. Blood from head and neck cancer patients was taken pre-exposure, 24 hr after the first fraction, 24 hr after the second fraction and 24 hr after the 25^th^ fraction. Blood from prostate cancer patients was taken pre-exposure, 48 hr after 1^st^ fraction, 24 hr after 5^th^ fraction (which was also the last fraction for these patients) and 1 month after the last fraction. For one prostate cancer patient blood was taken was taken pre-exposure, ½ hr after the first fraction, 24 hr after first fraction, 24 hr before the 5^th^/6^th^ fraction and 24 hr before the last fraction. For 4 breast and 2 lung cancer patients blood was taken was taken pre-exposure, ½ hr after the first fraction, 24 hr after first fraction, 24 hr before the 5^th^/6^th^ fraction and 24 hr before the last fraction. Blood from fluoroscopy patients was taken before treatment, 24 hr after treatment and 1 month after treatment. Blood from diagnostic CT patients was taken pre-exposure, 2 hr, 6 hr, 24 hr, 48 hr, 72 hr and 7 days after the diagnostic CT. Details of the fraction dose and calculated dose to the blood is given in Table 1. Blood was also taken from 39 normal blood donors for *ex vivo* storage experiments and blood was taken from three radiotherapy patients pre-exposure and 24 hr after radiotherapy treatment where cancer patients were irradiated with blood doses of 150 mGy for patient V, 140 mGy for patient VI and 80 mGy for patient VII for *ex vivo* versus *in vivo* experiments.

### Blood Dose Calculations

To determine the dose to the blood of patients undergoing radiotherapy, except TBI patients, the treatment planning system Eclipce (Varian, USA), which is a frequently used software in external beam planning, was used. This system uses the Anisotropic Analytical Algorithm to compute 3D dose distribution in patient volume. The software displays the geometry of the patient acquired by computed tomography and computed dose distribution. From these data for each patient determined irradiated volume (IV) was defined as a volume surrounded by five percent isodose and mean dose (Dmean) in this volume. Five percent Isodose is the surface that connects points in 3D dose distribution where the dose is equal to 5% of the prescribed dose. Doses lower than 5% of the prescribed dose can be neglected. Mean dose in the patient’s blood MBD was calculated according to the relation:

$${\rm{MBD}}={\rm{Dmean}}{\rm{.IBV}}/{\rm{BBV}}={\rm{Dmean}}{\rm{.}}({\rm{IV}}/{\rm{V}}){\rm{BBV}}/{\rm{BBV}}={\rm{Dmean}}{\rm{.IV}}/{\rm{V}}$$

where MBD - mean blood dose, Dmean – irradiated volume mean dose, IBV – irradiated blood volume, IV – irradiated volume, BBV – body blood volume and V – total patient volume (approximately equal to patient weight).

In this relation we assume that the blood in the patient’s body is irradiated homogeneously, blood is stored in the human body homogeneously and 1dm3 of human body weighs approximately 1 kg.

TBI patients blood dose was estimated from *in vivo* measurement on patient skin (9 measurement points) using thermoluminescent dosimeters. The blood dose of patients undergoing diagnostic CT was calculated using software ImPACT CTDosimetry (Medical Physics Department, St George’s Hospital, London, United Kingdom) and for the patients undergoing cardiac flouroscopy software PCXMX (Radiation and Nuclear Safety Authority, Helsinky, Finland) was used. Blood dose calculations are given in Table 1.

For cardiac fluoroscopy patients, the estimated effective dose is based on the following assumptions and is subject to significant uncertainty: 1. All the DAP data is assumed to be correct and in units of uGym2 or cGycm2 (these are the units that are reported by the Siemens equipment). 2. A single factor of 0.16 mSv/Gycm2 has been used to convert from DAP to effective dose. This factor is the factor for ‘complete coronary angio exam’ taken from table 15 in ‘Radiation Risks from Medical X-Ray Examinations as a Function of the Age and Sex of the Patient - CRCE-028’.

### Patient Blood Sampling

Blood samples were collected from the radiotherapy treated cancer patients in PAXGene tubes according to the manufacturer’s protocol (Qiagen, PreAnalytiX GmbH, Hilden, Germany). The tubes were kept at RT for 2 hr before being frozen at −20 °C. For comparison of *in vivo* and *ex vivo* irradiated samples, blood samples from radiotherapy patients were collected pre-exposure into heparin tubes. The samples were *ex vivo* irradiated and kept at 37 °C for 24 hr, after which they were injected into PAXGene tubes to be processed alongside the *in vivo* irradiated samples. RNA was extracted from the samples using the PAXGene Blood miRNA Kit (Qiagen, PreAnalytiX GmbH, Hilden, Germany) according to the manufacturer’s protocol. RNA quantity was assessed by Nanodrop ND2000 (Nanodrop, Wilmington, USA), and RNA quality was assessed by RIN values produced by Tapestation 2200 (Agilent Technologies, CA, USA).

### *Ex Vivo* Calibration Curve

Peripheral blood was taken from 10 healthy blood donors with informed consent and ethical approval from Berkshire Research Ethics Committee (reference number 09/HO505/87). 2–3 ml of peripheral blood were drawn from healthy human volunteers and filled into EDTA coated tubes (BD Biosciences, Heidelberg, Germany) with one tube per dose. Blood aliquots were irradiated with doses 0, 0.25, 0.5, 1, 2, 3, 4 Gy after which the samples were placed in an incubator at 37 °C for 24 hr. The irradiations were performed at room temperature with an A.G.O. HS X-ray system (Aldermaston, Reading, UK) (output 13 mA, 250 kV peak, 0.5 Gy/min).

### Multiplex Quantitative Real-Time PCR (MQRT-PCR)

*FDXR* expression in blood samples at all times points were analysed by MQRT-PCR. Reverse transcriptase reactions were performed using High Capacity cDNA Reverse transcription kit, (Applied Biosystems, FosterCity, CA, USA) according to the manufacturer’s protocol with 350 ng of total RNA. MQRT-PCR was performed using Rotor-Gene Q (Qiagen, Hilden, Germany). All reactions were run in triplicate using PerfeCTa® MultiPlex qPCR SuperMix (Quanta Biosciences, Inc. Gaithersburg, MD, USA) with primer and probe sets for target genes at 300 nM concentration each. 3′ 6-Carboxyfluorescein (FAM) and Texas Red (Eurogentec Ltd, Fawley, Hampshire, UK) were used as fluorochrome reporters for the hydrolysis probes analysed in multiplexed reactions with 2 genes per run including the control. Cycling parameters were 2 min at 95 °C, then 45 cycles of 10 s at 95 °C and 60 s at 60 °C. Data were collected and analyzed by Rotor-Gene Q Series Software. Gene target Ct (cycle threshold) values were normalized to a Hypoxanthine-Guanine phosphoribosyltransferase 1 (*HPRT1*) internal control. Ct values were converted to transcript quantity using standard curves obtained by serial dilution of PCR-amplified DNA fragments of each gene. The linear dynamic range of the standard curves covering six orders of magnitude (serial dilution from 3.2 × 10^−4^ to 8.2 × 10^−10^) gave PCR efficiencies between 93% and 103% for each gene with R^2^ > 0.998. Relative gene expression levels after irradiation were determined.

### Dose Estimation Curve

The mean expression level at each dose for these 10 donors was used to construct the dose estimation curve using a log transformed linear fit as previously described^17^. QPCR data was analysed using the delta CT approach. The Ct values of the unexposed were subtracted to create a correction factor, which was then subtracted from the *in vivo* Ct values. A correction factor was applied to the *in vivo* samples to account for differences between *in vivo* and *in vitro* samples. Doses were estimated using linear regression analysis (data not shown).

### Inter-lab Comparison MQRT-PCR

400 ng RNA from 0 hr and 24 hr samples were shipped on dry ice to lab 2. RNA was reverse transcribed (1×/25 °C/10 min, 1×/37 °C/120 min, 1×85 °C/5 min, 1 × 8 °C/10 min) with the High-Capacity cDNA Reverse Transcription Kit (Applied Biosystems (AB)). The resulting cDNA was diluted in a buffered solution and stored at −20 °C. The qRT-PCR was performed using TaqMan chemistry. After thawing and before using, the samples were heated up to 95 °C for 5 min (ThermoMixer, Eppendorf). TaqMan Universal Master Mix (AB), a specific primer-probe-design (AB) and water were mixed on ice and added to a 96-well plate. For each sample 15 ng of RNA equivalent was used in duplicates. The plate was sealed and centrifuged (1200 rpm, 1 min, Multifuge 3S-R, Heraeus). The following qRT-PCR run took place in our GeneAmp 7900 (AB) platform (1×/50 °C/2 min 1×/95 °C/10 min 40×/95 °C/1 min 60 °C/1 min). For a better comparability a manual threshold was used (0.05, baseline start: 6; end: 15). The cycle threshold (Ct) values of the genes of interest were normalized relative to 18S rRNA. For further analysis the fold change was calculated.

$${\rm{\Delta }}Ct=Ct(Gene\,of\,interest)-Ct(18S\,rRNA)$$

$${\rm{\Delta }}{\rm{\Delta }}Ct=Ct(Gene\,of\,interest)-Ct(Control)$$

$$Fold\,change={2}^{(-{\rm{\Delta }}{\rm{\Delta }}Ct)}$$

Every plate included a no template control and six standards for the calculation of a slope and run efficiency as quality control. The 18S rRNA-Ct values were used as quality control marker for the cDNA synthesis. The procedure was performed according to the standard operating procedures of the Bundeswehr Institute of Radiobiology (DIN EN ISO 9001/2008).

### Blood irradiated *ex vivo* and stimulated with LPS or curcumin

Peripheral blood from 10 healthy donors (5 men, 5 women; age range, 35–60 years) was collected into EDTA coated tubes and incubated with two different concentrations of LPS (1 ng/ml and 500 ng/ml) or curcumin (15 µM). Curcumin was prepared in DMSO (stock solution 10 mg/ml) and LPS in 50% ethanol (stock solution 1 mg/ml). LPS and curcumin were added to 500 µl of blood 1 hr before exposing it to 0 or 2 Gy X-rays (0.5 Gy/min) or just after exposure only for LPS. The blood samples were kept at 37 °C in an incubator with 5% CO_2_ for 2 hr and 24 hr after exposure to allow cells to undergo DNA repair. After the incubation time, the blood was mixed with 1 ml of RNAlater and stored at −80 °C until being processed for RNA extraction.

### Statistical Methods

Descriptive statistics were calculated in Minitab 17. Gene expression data were log2 transformed so that the data became normal distribution which represents a prerequisite for the employment of the parametric t-test. We also examined for equal variance of the compared group as another prerequisite of the t-test. Significant increases in FDXR expression was calculated a paired t-test with p < 0.05. A paired t-test was used to calculate the significant difference between patient samples accounting for variation among patients. Comparison of dose estimates was performed using a Mann Whitney test with p < 0.05. Linear regression analyses were performed using GraphPad Prism 7 software; R^2^ values are given. Data are presented as means ± standard error of mean (SEM). Linear regression modeling was performed with SigmaPlot 13.

### Data Availability

The datasets generated during and/or analysed during the current study are available from the corresponding author on reasonable request.

## Electronic supplementary material

Supplementary Information

Supplementary Dataset 1

Supplementary Dataset 2

Supplementary Dataset 3
