# Supplementary material for: FDXR is a biomarker of radiation exposure in vivo
Source: Sci Rep. 2018 Jan 12;8:684. doi: 10.1038/s41598-017-19043-w (PMC5766591; doi:10.1038/s41598-017-19043-w)
Supplement: Supplementary file 1 — Supplementary Information [file 41598_2017_19043_MOESM1_ESM.pdf]

FDXR is a biomarker of radiation exposure in vivo

Gráinne O'Brien[1], Lourdes Cruz-Garcia[1], Matthäus Majewski[2], Jakub Grepl[3,4,], Michael Abend[2], Matthias Port[2], Aleš Tichý[3,4], Igor Sirak[5], Andrea Malkova[6], Ellen Donovan[7], Lone Gothard[8], Sue Boyle[8], Navita Somaiah[8], Elizabeth Ainsbury[1], Lucyna Ponge[9], Krzysztof Slosarek[9], Leszek Miszczyk[9], Piotr Widlak[9], Edward Green[10], Neel Patel[10], Mahesh Kudari[10], Fergus Gleeson[10], Volodymyr Vinnikov[11], Viktor Starenkiy[11], Sergii Artiukh[11], Leonid Vasyliev[11], Azfar Zaman[12], Christophe Badie[1*]

[1]Public Health England, Centre for Radiation, Chemical and Environmental Hazards, Oxfordshire, U.K.

[2]Bundeswehr Institute of Radiobiology, Munich, Germany

[3]Department of Radiobiology, Faculty of Military Health Sciences in Hradec Králové, University of Defence in Brno, Czech Republic

[4]Biomedical Research Centre, Hradec Králové University Hospital, Czech Republic

[5]Department of Oncology & Radiotherapy and 4th Department of Internal Medicine - Hematology, University Hospital, Hradec Králové, Czech Republic

[6]Department of Hygiene and Preventive Medicine, Faculty of Medicine in Hradec Králové, Charles University, Czech Republic

[7]Centre for Vision, Speech and Signal Processing, University of Surrey, Guildford, GU2 7TE, U.K;

[8]Institute of Cancer Research / Royal Marsden NHS Foundation Trust, Downs Road, Sutton SM2 5PT, U.K.

[9]Maria Sklodowska-Curie Institute – Oncology Center, Gliwice Branch, Gliwice, Poland.

[10]Department of Radiology, Churchill Hospital, Oxford, U.K.

[11]Grigoriev Institute for Medical Radiology, National Academy of Medical Science, Kharkiv, Ukraine

[12]Department of Cardiology, Freeman Hospital and Institute of Cellular Medicine, Newcastle University, Newcastle-upon-Tyne, UK.

Supplementary Table S1

MQRT-PCR data from Figure 2. Ct values, starting quantity values (initial copy number of template) and basal level of expression (normalization of gene of interest to the housekeeping gene) for housekeeping gene *HPRT* and *FDXR* are provided in triplicate for peripheral blood samples from (A) TBI, (B) endometrial cancer, (C) prostate cancer, (D) lung cancer, (E) head & neck cancer, (F) breast cancer, (G) cardiac fluoroscopy and (H) CT scan patients as detailed in Table 1.

Supplementary Table S2

MQRT-PCR data from Figure 3. Ct values, starting quantity values (initial copy number of template) and basal level of expression (normalization of gene of interest to the housekeeping gene) for housekeeping gene *HPRT* and *FDXR* are provided in triplicate for (A) peripheral blood irradiated ex vivo with doses 0 Gy, 0.25 Gy, 0.5 Gy, 1 Gy, 2 Gy, 3 Gy and 4 Gy from 10 normal donors, (B) peripheral blood samples at 0 hr in 82 healthy human donors and in blood kept ex vivo at 37°C for 24 hr in 39 healthy human donors, and (C) for blood irradiated *ex vivo* and *in vivo* from three donors (I, II and III).

Supplementary Table S3

MQRT-PCR data from Figure 5. Ct values, starting quantity values (initial copy number of template) and basal level of expression (normalization of gene of interest to the housekeeping gene) for housekeeping gene *HPRT* and *FDXR* are provided in triplicate for peripheral blood irradiated and/or stimulated with LPS and curcumin *ex vivo*.
